# Addressing indirect sourcing in zero deforestation commodity supply chains

**DOI:** 10.1126/sciadv.abn3132

**Published:** 2022-04-29

**Authors:** Erasmus K. H. J. zu Ermgassen, Mairon G. Bastos Lima, Helen Bellfield, Adeline Dontenville, Toby Gardner, Javier Godar, Robert Heilmayr, Rosa Indenbaum, Tiago N. P. dos Reis, Vivian Ribeiro, Itohan-osa Abu, Zoltan Szantoi, Patrick Meyfroidt

**Affiliations:** 1 Georges Lemaître Earth and Climate Research Centre, Earth and Life Institute, UCLouvain, Bâtiment Mercator, Place Louis Pasteur 3, B-1348 Louvain-la-Neuve, Belgium.; 2 Fonds de la Recherche Scientifique F. R. S.-FNRS, Rue d’Egmont 5, B-1000 Brussels, Belgium.; 3 Stockholm Environment Institute, Linnégatan 87D, 115 23 Stockholm, Sweden.; 4 Global Canopy, 3 Frewin Court, Oxford OX1 3HZ, UK.; 5 European Forest Institute, Sant Pau Historic site, Sant Leopold Pavillon, St. Antoni M. Claret, 167, 08025 Barcelona, Spain.; 6 Environmental Studies Program and Bren School of Environmental Science, University of California, Santa Barbara, Santa Barbara, CA 93106, USA.; 7 Julius-Maximilians-University of Würzburg, Institute for Geography and Geology, Department of Remote Sensing, Oswald-Külpe-Weg 86, 97074 Würzburg, Germany.; 8 European Space Agency, Directorate of EO Programmes, Science, Applications and Climate Department, Frascati 00044, Italy.; 9 Stellenbosch University, Stellenbosch 7602, South Africa.

## Abstract

The trade in agricultural commodities is a backbone of the global economy but is a major cause of negative social and environmental impacts, not least deforestation. Commodity traders are key actors in efforts to eliminate deforestation—they are active in the regions where commodities are produced and represent a “pinch point” in global trade that provides a powerful lever for change. However, the procurement strategies of traders remain opaque. Here, we catalog traders’ sourcing across four sectors with high rates of commodity-driven deforestation: South American soy, cocoa from Côte d’Ivoire, Indonesian palm oil, and Brazilian live cattle exports. We show that traders often source more than 40% of commodities “indirectly” via local intermediaries and that indirect sourcing is a major blind spot for sustainable sourcing initiatives. To eliminate deforestation, indirect sourcing must be included in sectoral initiatives, and landscape or jurisdictional approaches, which internalize indirect sourcing, must be scaled up.

## Teaser

Indirect sourcing by commodity traders is a blind spot for efforts to eliminate commodity-driven deforestation.

## INTRODUCTION

The trade in agricultural commodities—products including cotton, coffee, cocoa, grains, oilseeds, and livestock—is a mainstay of the global economy, providing food, fuel, and fiber to consumers around the world. The long, complex supply chains that process and transport these products bridge the gap between producers and consumers but are criticized for masking negative socioenvironmental impacts and obfuscating the allocation of responsibility for these impacts. A particular concern for commodities produced in the tropics is habitat destruction—the loss and degradation of forests and other natural ecosystems to expanding agriculture. Almost one-third of forest loss is driven by commodity production (*1*). Many companies have made zero deforestation commitments, although progress in implementing deforestation-free supply chains remains slow (*1*, *2*).

Commodity traders (or simply “traders”) have a key role in achieving deforestation-free sourcing. Traders, including multinationals such as Cargill, Olam, and Bunge, as well as many smaller companies, handle the procurement, processing, and export of commodities from producer to consumer countries. Downstream companies (i.e., companies operating closer to consumers and further from farmers) ultimately rely on traders to implement their sustainable sourcing commitments on the ground. Commodity trading is consolidated, creating a “pinch point” in global supply chains, with a small number of traders handling most of the world’s trade in each product. This market concentration creates an opportunity where a small number of companies have the leverage to improve the sustainability of a large proportion of the world’s sourcing (*3*). Although traders are sometimes considered a “missing” or “hidden” link in supply chain governance (*4*), there is increasing emphasis on their role. At the 2021 United Nations (UN) Climate conference (COP-26), 10 of the world’s largest commodity traders published a “shared commitment to halting forest loss,” and the European Union has published proposed legislation that requires traders to “submit a due diligence statement… thereby becoming liable for compliance of the relevant commodity,” i.e., imposing a legal responsibility for trading companies to ensure their sourcing is not linked to deforestation (*5*). As of January 2022, Germany and France have already passed legislation imposing new due diligence requirements, with similar bills focused on deforestation also being considered in the United Kingdom and United States.

Despite the importance of traders’ actions for sustainable procurement efforts, few studies address how traders operate in countries of production (*4*, *6*). The academic literature on sustainable supply chains is instead focused on apparel, automotive, chemical, electronics, and retail companies (*7*, *8*), and where studies do analyze traders’ sourcing, they use varying terminology (table S1) and focus on single sectors (*2*, *9*–*12*), limiting learning across contexts.

In this study, we present a framework distinguishing traders’ “direct” sourcing from producers (i.e., farmers) and “indirect” sourcing via local intermediaries. Then, for four deforestation risk contexts (South American soy, cocoa from Côte d’Ivoire, Indonesian palm oil, and Brazilian live cattle exports), we assemble data—including detailed shipment data, corporate reports and disclosures, facility processing capacities, animal movement records, and farm production data—to quantify the sourcing strategies of trading companies covering the top 60% of trade in each context. We reveal that indirect sourcing is a widespread and often dominant strategy, and we review why it is so common. We then document how corporate sustainable procurement strategies largely ignore indirect sourcing, undermining progress on zero deforestation goals. Last, we discuss ways of achieving more progress on zero deforestation commodity production, given the reality that large parts of the supply chain are and will continue to be indirectly sourced via local intermediaries. There are no silver bullets, but to deliver on promises to eliminate deforestation from commodity supply chains, efforts for sector-wide transparency and jurisdictional sourcing must be scaled up and combined with other sustainable procurement approaches.

### Direct and indirect sourcing

**Fig. 1.**
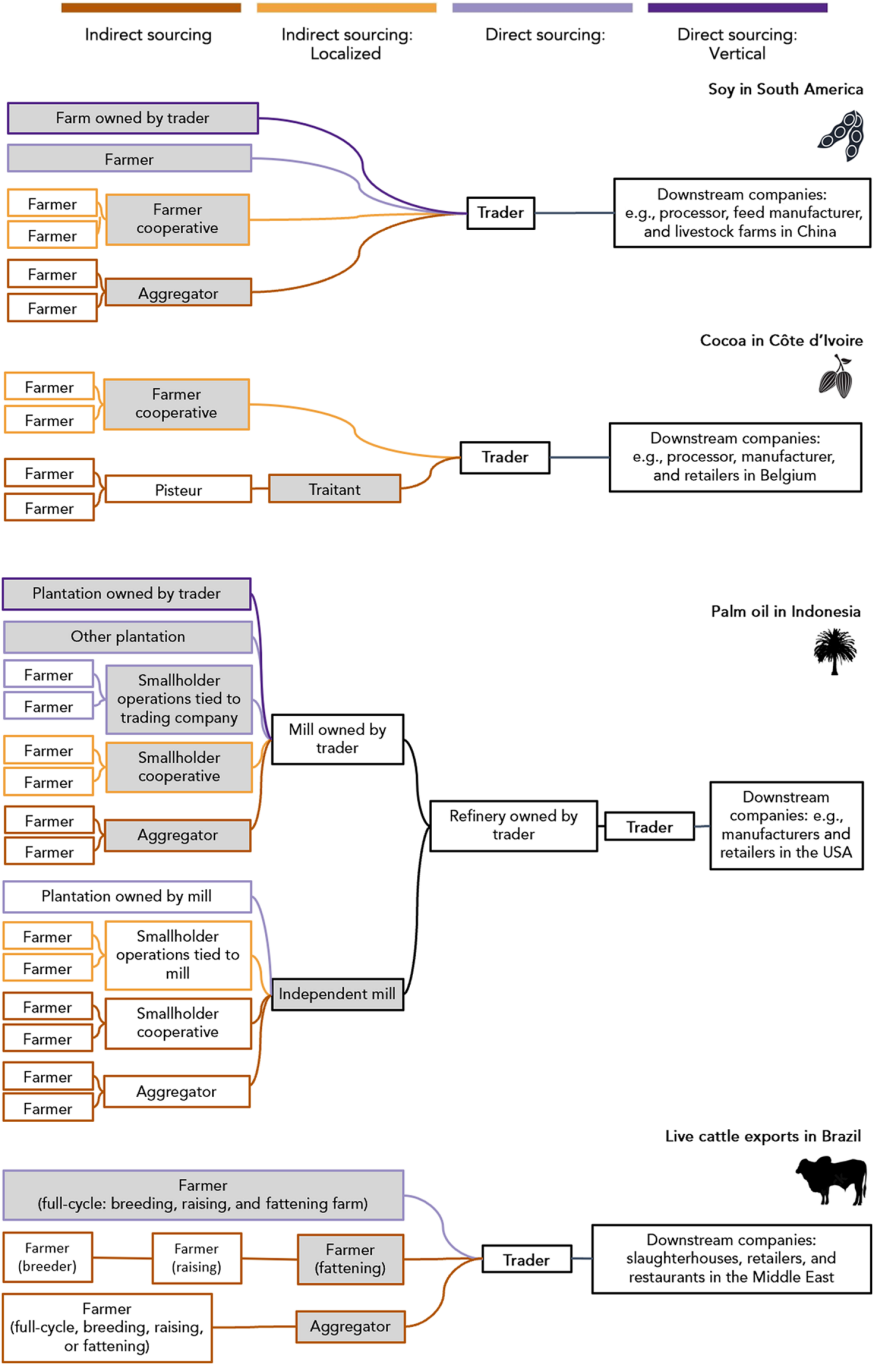
The supply chains of our four focal commodity contexts. Gray boxes are a trader’s direct suppliers. Traders’ direct sourcing is shown in purple, and indirect sourcing is shown in orange/brown; black lines can contain a mix of both direct and indirect sourcing. Direct sourcing: vertical (dark purple) refers to vertically integrated traders who operate their own farms. Supply chains are simplified to showcase the different sourcing strategies and intermediaries. Soybeans, for example, may be crushed into soybean meal and oil before export, and farmers may own multiple farms.

Sustainability risks can occur at any level of the supply chain, but in agricultural commodity supply chains, they are usually concentrated at the location of production. Deforestation, other forms of ecosystem degradation, and negative social impacts such as child or forced labor occur more commonly on farms rather than higher up among intermediaries in the supply chain. We therefore classify traders’ sourcing strategies by making a key distinction between products that trading companies source “directly” from producers versus “indirectly” from other intermediaries (e.g., brokers and aggregators) in the supply chain. Without taking active steps to overcome information asymmetries, companies inevitably have less information about the origin of products sourced indirectly rather than directly. The terms “direct sourcing” and “indirect sourcing” are commonly referred to in industry documents and academic research but not used consistently across contexts (table S1). We propose the following frameworks (Fig. 1):

1) A direct supplier (sometimes referred to as a “tier-1” supplier) is the actor in the supply chain from whom a company purchases and takes physical control or “custody” over a product.
2) Indirect suppliers are all the actors in the supply chain that are more than one tier removed from the company (i.e., a direct supplier’s own suppliers).
3) Direct sourcing is when the company procures a product from the original producer, i.e., the supply chain does not involve indirect suppliers. In vertically integrated supply chains, the focal company produces the commodity themselves.
4) Indirect sourcing is procurement from a supply chain that includes indirect suppliers. Indirect sourcing can sometimes be localized to a specific geography. If the intermediary has a geographic location (e.g., a farmer cooperative or a storage company operating a silo), then the raw product can be assumed to come from the surrounding landscape. This knowledge of the approximate location of commodities can be valuable for the purposes of assessing and triaging sustainability risks in supply chains.

### Deforestation risk commodities

From 2001 to 2015, cattle, palm oil, soy, and cocoa production were the four leading drivers of commodity-driven deforestation (*13*). We analyzed the sourcing strategies of trading companies across the leading producing regions of each commodity, described below.

#### South American soy

**Fig. 2.**
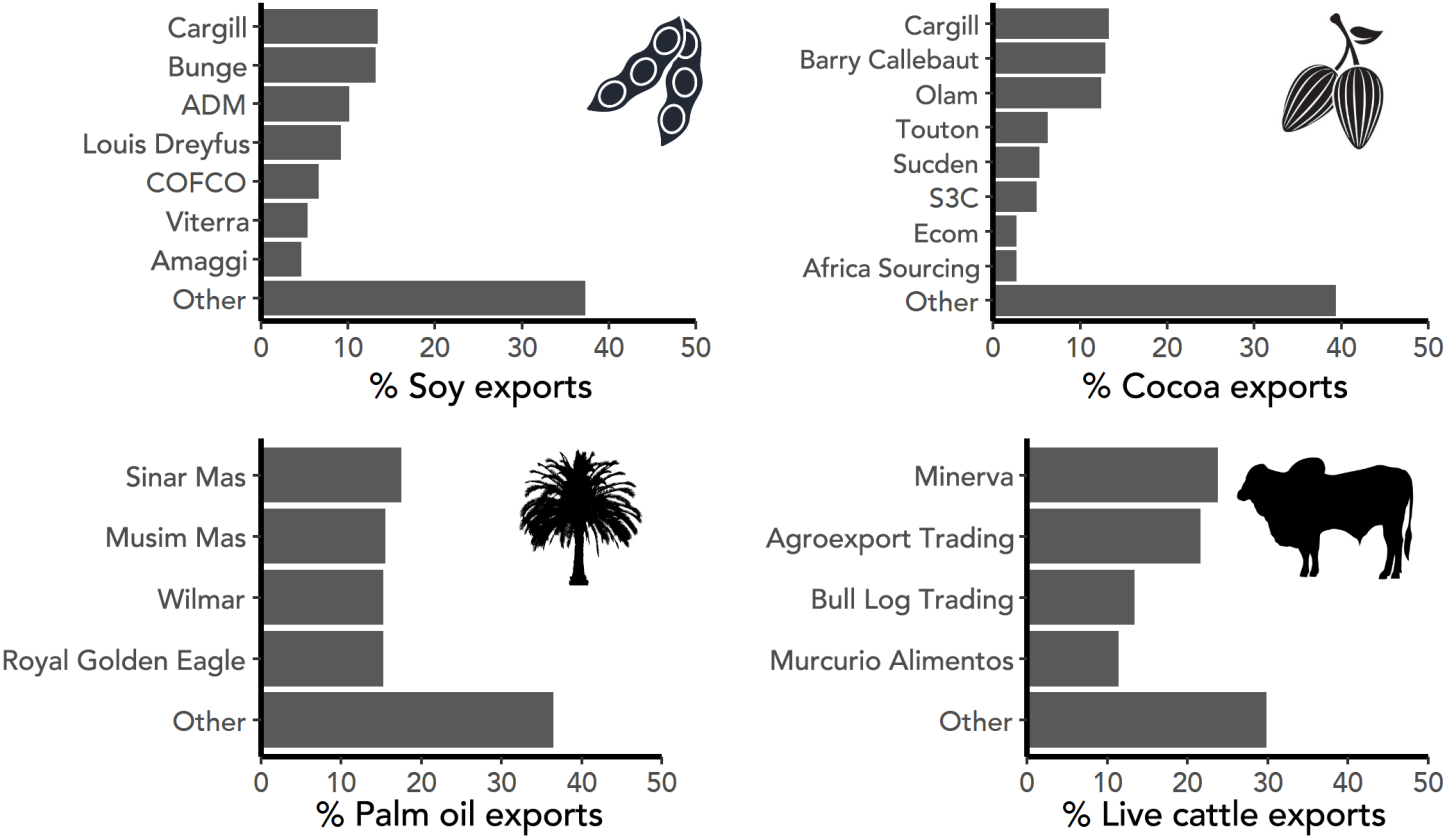
Market share of trading companies. Traders responsible for the top 60% of exports across our four focal contexts: South American soy, cocoa from Côte d’Ivoire, Indonesian palm oil, and Brazilian live cattle.

Together, Brazil, Argentina, and Paraguay produce more than half of the world’s soy (*14*). The harvested area in these three countries more than tripled in the past 30 years to cover more than 50 Mha (*14*). As a result of this expansion, soy has been both a direct and indirect driver of deforestation across the Brazilian Amazon and Cerrado, and the Paraguayan and Argentinian Chaco (*15*). In 2018, seven trading companies (Cargill, Bunge, Archer Daniels Midland, Louis Dreyfus, COFCO, Viterra, and Amaggi) handled 62.5% of exports from the region (Fig. 2). Most soy is produced by large-scale producers [farming areas of more than 1000 ha (*16*)], who may operate their own silos (grain storage facilities) or have soy production part-financed by trading companies. Soy traders may either have direct contracts with soy producers, buy from groups of farmers (cooperatives) who operate a shared silo, or accept delivery from aggregators who, in turn, buy from a variety of farmers, with sourcing switching depending on the season (Fig. 1).

#### Cocoa from Côte d’Ivoire

In Côte d’Ivoire, hundreds of thousands of smallholder farmers produce 45% of the world’s cocoa harvest (*14*). Cocoa makes up more than 20% of Côte d’Ivoire’s export revenues, although the sector is troubled by persistent poverty, child labor, and deforestation. Forest cover in Côte d’Ivoire fell from 15% in 1986 to 9% in 2015, in large part because of the expansion of cocoa (*17*). In 2019, eight companies (Cargill, Barry Callebaut, Olam, Touton, Sucden, S3C, Ecom, and Africa Sourcing) handled 60.6% of exports (Fig. 2). In practice, all of their cocoa sourcing is indirect, as cocoa is not purchased from smallholder farmers directly but from cooperatives or other middlemen: local “pisteurs” who visit villages collecting cocoa before selling on to larger-scale local intermediaries, “traitants” (Fig. 2). Despite efforts by the coffee and cocoa board [Conseil Café Cacao (CCC)] to register pisteurs and traitants, many work unlicensed. From the perspective of monitoring supply chain risks, cocoa sourced from cooperatives is localized, in that it comes from the region where the cooperative has members. In contrast, cocoa bought from traitants (often exchanged at the port) may have a completely unknown origin.

#### Indonesian palm oil

Indonesia produces 60% of the world’s oil palm fruit, fueled through recent rapid expansion: Between 1995 and 2015, 450,000 ha of new plantations were established each year, driving more than 100,000 ha year^−1^ of deforestation (*18*). In 2018, four companies (Sinar Mas, Musim Mas, Wilmar, and Royal Golden Eagle) handled 64% of exports. Palm oil flows from plantations (which may be smallholder or industry-owned production) to local mills, refineries, and traders. Thirty-four percent of oil palm fruit in Indonesia is produced by smallholder farmers (*19*). Smallholders may contract their land to plantation companies, or they may produce palm fruits as part of a company scheme (also known as “plasma schemes”) selling to a specific company’s mills. Smallholders may operate independently or organize themselves into cooperatives. Independent smallholders can themselves sell to local mills, although most sell via local aggregators who then sell to mills (*20*). Traders may operate mills and refineries themselves, although most of the mills are independent—also known as “third-party” mills.

#### Brazilian live cattle exports

The Brazilian cattle sector is the leading driver of tropical deforestation. Brazil is the world’s largest exporter of cattle products (including beef, offal, and live cattle), although here we focus specifically on the live cattle trade because it is comparatively understudied while being a hot spot of deforestation. Brazil exports 200,000 to 790,000 live cattle per year, with 85% of exports (2010–2019) originating from the Amazon state of Pará. Because of their concentration in the Amazon, live cattle exports are linked to 11.6% of deforestation risk, although they make up only 3.9% of cattle exports from Brazil by value (*12*). In 2019, four companies, Minerva Global Foods (henceforth “Minerva”), Agroexport Trading, Bull Log Trading, and Mercúrio Alimentos, handled 70.2% of exports (Supplementary Text). While some “full-cycle” ranches rear cattle from birth to export (or slaughter), cattle are often moved multiple times in their lifetime (Fig. 2). They may be born on one farm, reared on another, and fattened on a third before being exported. In these cases, the property selling to the trader (the direct supplier) is only the last tier in the supply chain from birth to export.

## RESULTS

### Commodities are commonly sourced from local intermediaries by traders

**Fig. 3.**
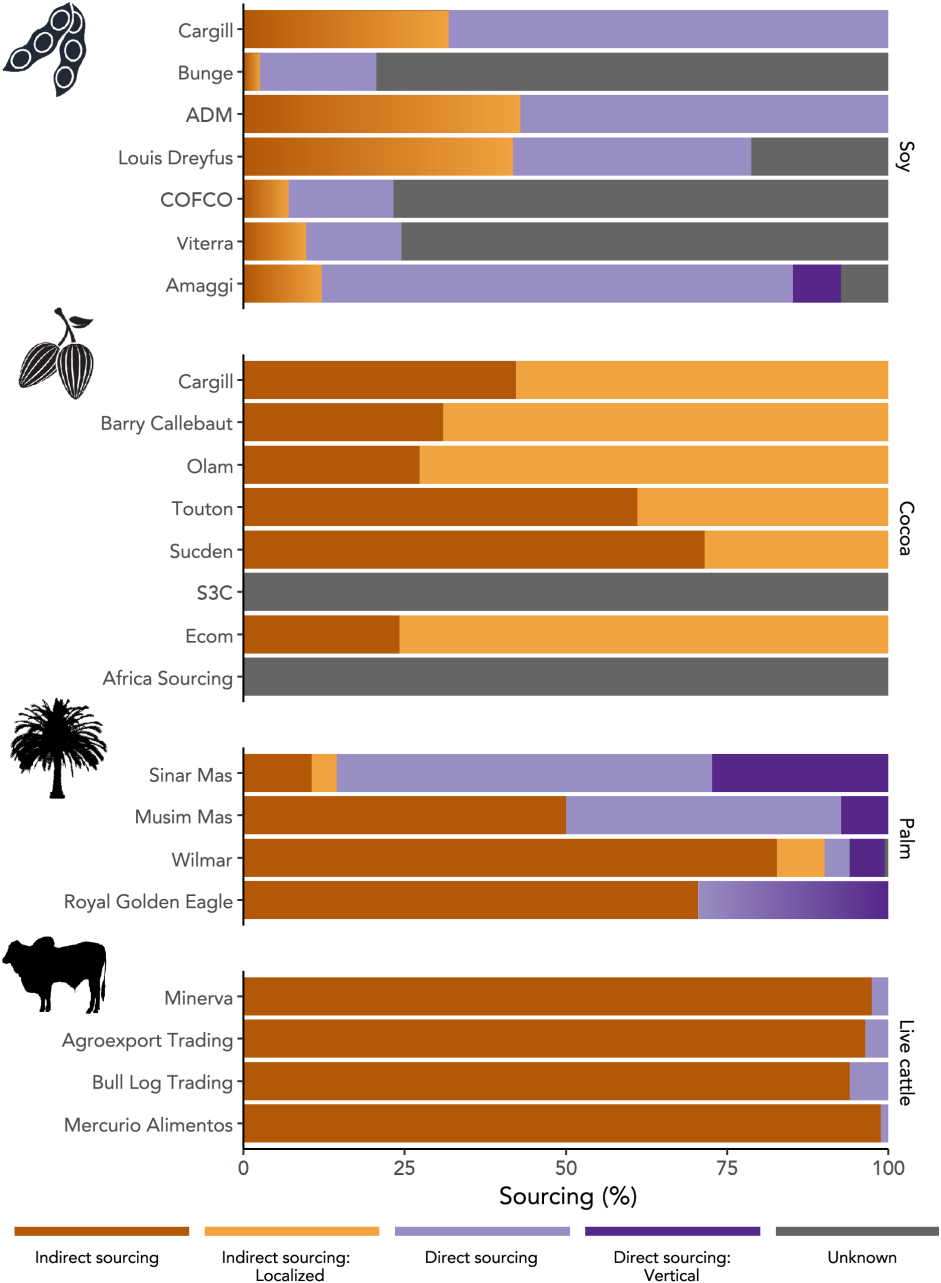
The prevalence of different sourcing strategies. The proportion of commodities that are indirectly (orange) and directly (purple) sourced by major traders exporting soy from South America, cocoa from Côte d’Ivoire, palm oil from Indonesia, and live cattle from Brazil. We use a color gradient to portray uncertainty in the different types of indirect sourcing used by soy traders and the different types of direct sourcing used by Royal Golden Eagle (see Materials and Methods).

Where we were able to differentiate direct and indirect sourcing for more than half of a trader’s supply chain, we found that indirect sourcing made up 12 to 42% of soy sourcing, 15 to 90% of palm oil sourcing, 94 to 99% of live cattle exports, and 100% of cocoa sourcing (Fig. 3). Agricultural commodity supply chains have multiple tiers already within producer countries, with each direct supplier, in turn, buying from dozens to hundreds of indirect suppliers. In Côte d’Ivoire’s cocoa sector, for example, Barry Callebaut’s cooperatives included a median 504 member farms [interquartile range (IQR): 359 to 826; range: 151 to 3463]. Each of Minerva’s direct suppliers, in turn, bought cattle from a median of 20 other properties (IQR: 7 to 59; range: 1 to 1334).

In terms of traders’ understanding of the ultimate origin of their sourcing, we highlight the difference between products sourced through farmer cooperatives (localized indirect sourcing) versus other brokers and aggregators. For palm oil and cocoa, respectively, 4 to 7% and 29 to 76% of each trader’s sourcing was localized, in that it was sourced from farmer cooperatives, which can be mapped in space to give an approximate origin for products (Fig. 3).

### What drives indirect sourcing?

Historically, there has been little pressure for traders to source directly from or identify the original producers of commodities. By definition, a commodity is a basic good that is “interchangeable with other products of the same type” (*21*). Commodity exchanges and trade associations, such as the Chicago Board of Trade, São Paulo B3, and the Cocoa Association of London, have been developed to maximize this interchangeability. Commodities (and their contracts) are classified into standardized grades based on product characteristics (*22*). Soybean contracts, for example, will list acceptable ranges for each shipment’s oil content, moisture content, contamination with other materials (“foreign matter”), and the percentage of damaged beans or green beans. These grading systems, which do not include information about sustainability, give traders flexibility to fulfill commodity contracts using products of any origin but of the same grade, as dictated by price and logistics.

This fungibility is particularly apparent in spot markets, where traders make short-term, opportunistic trades. The sourcing of cattle by traders, for example, operates almost exclusively as a spot market, as farmers rarely sign long-term supplier contracts. In the palm oil sector, traders often commit to purchases over periods of 3 to 9 months from a given direct supplier (*10*), although palm oil refineries and traders also make opportunistic purchases (“spot trades”). These purchases may be used to top up volumes when supply drops below a facility’s target or to take advantage of palm oil that becomes available at a low price. In these cases, the origin of the palm oil is a secondary consideration and information about the origin (e.g., the supplier list) is commonly not provided until after a purchase is made (*10*).

Moreover, there is a strong economic logic for traders to source via local intermediaries. Intermediaries reflect specialization in the supply chain. Local middlemen specialize in product sourcing and aggregation, while traders specialize in the logistics of export, international trade, and other aspects of commodity trading, including speculation on commodity futures and currency movements, arbitrage, and investing in financial instruments and private equity (*23*). For example, the variation in reliance on indirect sourcing among palm oil traders (Fig. 2) reflects differences in business strategy. Companies, such as Wilmar, which have invested heavily in palm oil refinery capacity and focus on the export of higher-value products such as refined palm oil have a greater reliance on indirect sourcing than companies, such as Sinar Mas, which are focused on growing, milling, and exporting crude palm oil (*11*, *23*). In the cattle sector, production is further fragmented—farmers specialize in the production of calves, calf-rearing, or fattening, which introduces extra intermediaries between the location where cattle are born and traders’ direct suppliers (Fig. 2). For traders, intermediaries who aggregate products from hundreds or thousands of individual producers thus help reduce their transaction costs. While still a substantial share of the market, the level of indirect sourcing is notably lower for soy supply chains, where farming is often large scale, than for oil palm, cocoa, and cattle, which commonly come from smaller, less capitalized producers. It would be more costly for traders to form individual contracts with hundreds of thousands of farmers than to buy from a smaller number of local intermediaries who each aggregate supply from multiple farms. In some cases, the physical characteristics of the commodity lend itself to aggregation early in the supply chain. Fresh oil palm fruit bunches are perishable and must be processed within 48 hours to avoid spoilage. They are therefore aggregated and processed locally before long-distance distribution in the more stable form of crude palm oil. Last, from the farmer’s perspective, they may also gain market power when selling as a group through a cooperative compared to individually negotiating contracts with traders—a critical concern when considering the imbalanced power dynamics between multinational purchasing companies and individual farmers.

### The challenge that indirect sourcing poses to zero deforestation commodity sourcing

It is simpler for companies to identify, engage with, and exert influence over their direct suppliers, with whom they have contractual relations, than actors more than one tier removed from them (*7*, *24*). Mirroring this market reality, trading companies have been slower to engage with and take responsibility for the actions of their indirect suppliers. In 2009, under civil society pressure, major Brazilian meat-packers, including Minerva (which both slaughters and exports cattle), committed to zero deforestation in their supply chains in the Amazon biome. As a result of these so-called “Cattle Agreements,” meat-packers implemented systems for monitoring their direct suppliers. Over the following years, the blind spot of indirect suppliers was highlighted repeatedly (*25*, *26*), although it was not until 2020 that Minerva and other companies in the sector announced that they would expand monitoring to include indirect suppliers in the Amazon and Cerrado biomes (*27*). Key details of this monitoring remain unclear, including the mechanisms of implementation and cutoff dates. In the soy sector, Cargill is mapping the location of direct supplier farms, but for indirectly sourced soy, it so far reports only mapping the points of procurement (i.e., farmer cooperatives or silos) (*28*). In some cases, companies explicitly exclude their indirect suppliers from sustainable sourcing commitments. COFCO, the fifth largest soy trader, specifies that its sustainable sourcing policy applies only to direct suppliers (*29*). Similarly, cocoa industry efforts to increase traceability do not include indirect sourcing through traitants. The Cocoa & Forests Initiative (CFI), a multi-stakeholder initiative signed by the governments of Côte d’Ivoire and Ghana and leading cocoa companies, is a case in point. Companies participating in the CFI, including eight trading companies active in Côte d’Ivoire, have set targets for mapping cocoa sourced via cooperatives but are notably silent about mapping or addressing sustainability risks in cocoa sourced via other intermediaries (*30*), which makes up 20 to 70% of all cocoa sourced by each trader (Fig. 2).

**Fig. 4.**
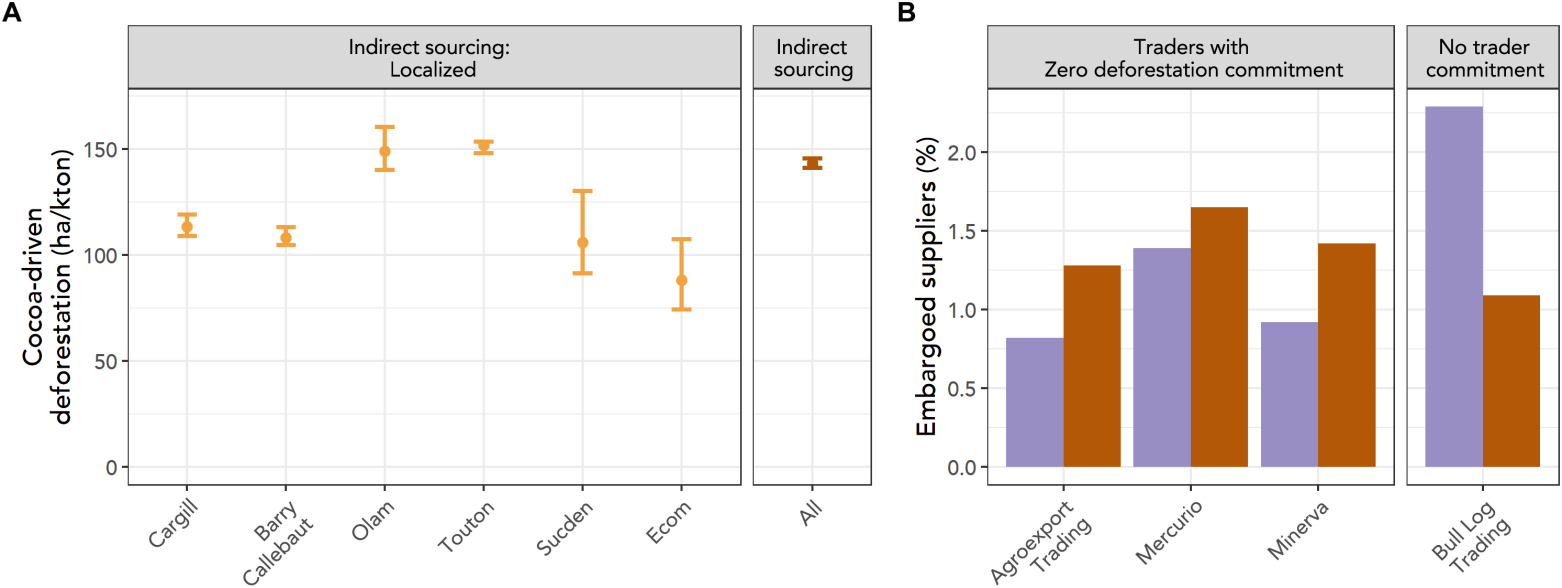
Deforestation risks are higher in the parts of the supply chain over which companies have the least visibility. (**A**) For four of six cocoa traders whose sourcing through cooperatives (localized indirect sourcing) is known, deforestation risks (ha/kton cocoa) are higher in the indirect supply chain. Error bars are 95% confidence intervals, reflecting uncertainty in the estimation of volumes sourced by each trader from each cooperative (Supplementary Text). (**B**) Embargoed properties are more common among indirect (orange) than direct (purple) suppliers for live cattle traders that have adopted zero deforestation commitments; the opposite pattern holds for Bull Log Trading, which had not made a commitment.

Unfortunately, deforestation and related risks are often higher in precisely the parts of the supply chain over which companies have the least visibility (Fig. 4). In the Côte d’Ivoire cocoa sector, for example, we mapped localized indirect sourcing through cooperatives for six traders. For four of these six, the relative deforestation risk (hectares/kiloton of cocoa) of cocoa sourced through cooperatives (“localized indirect”) was lower than for the remaining cocoa, sourced indirectly through traitants (Fig. 4A). In the Brazilian cattle sector, indirect suppliers to slaughterhouses with zero deforestation commitments are 1.42 times more likely to deforest than direct suppliers (*31*). Deforestation risks among indirect suppliers also translate into hidden legal risks. A study of beef exports from the Brazilian states of Mato Grosso and Pará found that 48% of exports were contaminated with potentially illegal deforestation when considering meat-packers’ indirect suppliers—in comparison with 12% contamination when looking only at their direct suppliers (*32*). A similar pattern emerges when evaluating the risk of purchasing cattle from properties with confirmed cases of illegality: In Brazil, properties with noncompliant deforestation may be placed on a list of embargoed areas, from which it is not permitted to purchase goods. We assessed live cattle traders’ cattle purchases and found that, for companies who have adopted zero deforestation commitments, embargoed areas are 1.2 to 1.6 times more common among indirect than direct suppliers (Fig. 4B and Supplementary Text).

Indirect suppliers can pose higher deforestation risks through several pathways (Supplementary Text). Of particular concern, however, is that a focus on direct suppliers creates leakage, where noncompliant production is not eradicated but displaced from direct to indirect suppliers. After meat-packers began monitoring their direct suppliers in the Brazilian Amazon, the cattle ranches that they bought directly from did reduce deforestation (*26*). Laundering of cattle from noncompliant indirect suppliers to compliant direct suppliers is, however, widespread (*9*, *25*), and the meat-packers’ commitments have had little effect on overall deforestation rates (*33*). Similarly, one of the success stories of corporate environmental efforts, the Soy Moratorium is also at risk of being undermined by unverified indirect sourcing (Supplementary Text). The Soy Moratorium is a multi-stakeholder landscape initiative to avoid the purchase of soy planted on recently deforested land in the Amazon. Between 2006 and 2016, the moratorium is estimated to have avoided 18,000 ± 9000 km^2^ of deforestation (*34*), although compliance is still not 100%. In 2018, 882.34 km^2^ of soy were planted on land cleared after the moratorium cutoff date, making up 4.8% of the deforestation in the monitored area (*35*). A key mechanism through which noncompliant soy reaches the market is indirect sourcing, as only 45% of traders specify cutoff dates and compliance with the Moratorium in contracts for their indirectly sourced soy (*36*).

## DISCUSSION

### Prospects for eliminating deforestation from commodity supply chains

Here, we have focused on one part of agricultural commodity supply chains, from farmers to traders, showing that (i) indirect sourcing via local intermediaries is common, (ii) traders have been slow to address their indirect sourcing, and (iii) sustainability risks are often higher through indirect sourcing. These three observations, when taken together, expose systemic limitations of current sustainable procurement efforts. Below, we identify six approaches (Table 1) that traders currently adopt, to varying degrees, to ensure their sourcing complies with sustainability commitments. This list is not intended to be exhaustive or definitive; we focus on common approaches and assess how each addresses the challenge of ensuring sustainability in indirectly sourced products. These different sustainable procurement approaches are interdependent and not used in isolation. Certification can be implemented at multiple levels—at farms, cooperatives, or landscapes; companies may source through a landscape initiative while also requiring traceability and cascading compliance from their tier-1 suppliers; and there can also be no transparency of supply chain connections without first establishing traceability.

**Table 1.** Common approaches taken by companies to ensure that sourcing complies with sustainability commitments. This includes a summary of how each approach addresses risks in indirect sourcing as well as their strengths and weaknesses in delivering more sustainable commodity sourcing.

**Sustainable** **sourcing strategy**	**How addresses risks in indirect** **sourcing**	**Strengths**	**Weaknesses**	**Interdependencies**	**Examples**
Increased direct sourcing	Reduces indirect sourcing	Reduces transaction costs and information asymmetries as focal company has direct contract with (or owns) the properties producing raw materials (*44*)	Increases market concentration; can exclude local actors; may increase costs if companies outstretch their core competencies; impact of sustainable procurement efforts are limited to own supply chain; can result in bifurcated supply chains if buyers shift supply to low-risk regions	Requires transparency or certification of on-farm activities (e.g., zero deforestation) to be externally demonstrable	Between 2018 and 2019, the soy trader Viterra increased direct sourcing in “priority municipalities” in Brazil’s Matopiba region from 42.9 to 64.9% (*88*, *89*)
Cascading compliance	Requires direct suppliers to, in turn, engage with their suppliers and communicate sustainable procurement requirements	Low cost for focal company (*41*)	Passes burden of enforcing compliance to actors with (in many cases) lower capacity (*41*); impact remains limited to actors in own supply chain	Requires transparent third-party audits or certification to be externally demonstrable (*41*)	Few examples by agricultural commodity traders; more commonly used by downstream companies, e.g., Consumer Goods Forum Forest Positive Coalition of Action members that pass compliance requirements onto traders and other suppliers (*77*)
Certification	Provides evidence that producer meets certification standard without requiring the trader to monitor or have direct relationship with producers	Recognized by consumers; downstream companies do not necessarily need to identify product origin when buying certified products; mass-balance or book-and-claim approaches keep costs low	Low additionality (*48*); risks market bifurcation; book-and-claim and mass-balance approaches hinder traceability and create disconnect between product labels and content (*90*); low transparency leads to questions over audit quality	Can be implemented at multiple scales (farm, cooperative, or landscape), though usually implemented at farm or cooperative level	Rainforest Alliance/UTZ and Fairtrade for cocoa, RSPO for palm oil, RTRS for soy
Traceability	Seeks to track all sourcing back to farm	Increases focal company’s understanding of their supply chain and sourcing risks; makes engagement between trader and producers possible (e.g., around zero deforestation requirement, preferable contracts, and market access)	Requires segregation for commodities that are traditionally stored and processed in bulk; because of costs, often limited to direct supply chains only or to “high-risk” regions (*57*); where optional, it risks market bifurcation; doubts over reliability of some farm-level mapping exercises (*9*, *56*)	In itself, traceability does not affect the mode of production of a commodity; thus requires cascading compliance or communication of sourcing standards to induce a change at the farm level; requires transparency or certification for sustainability claims to be externally demonstrable	Between 2019 and 2020, the oil palm trader Musim Mas increased traceability back to the plantation from 49 to 66% (*53*); in 2020, Minerva monitored 3770 direct suppliers in Brazil for deforestation (*91*)
Transparency	Open communication of progress (e.g., on traceability, risks, implementation measures, and outcomes) and challenges implementing sustainable procurement practices in direct and indirect sourcing	Allows companies to communicate with consumers about product origins and demonstrate progress against sustainability targets; discourages greenwashing and malpractice (*61*)	Data disclosures are nonstandardized; where optional, it risks market bifurcation (*61*); limited by data availability (e.g., may only focus on the direct supply chain)	A prerequisite for transparency is knowledge of both the focal company’s supply chain (e.g., through traceability, certification, or information on suppliers) and on-the-ground impacts (e.g., through independent or own satellite monitoring) (*61*)	Leading cocoa traders have disclosed which cooperatives they source from in Côte d’Ivoire (*55*)
Landscape approaches	Internalizes all production practices—among both direct and indirect suppliers—within the landscape where commodity originates	The only approach that captures land use dynamics outside of monitored supply chains; seeks to build capacity and identify solutions across sectors (*74*); avoids redundancy between overlapping certification and corporate sustainability programs; jurisdictional sourcing efforts reward jurisdictions that implement sustainable land use plans (*75*)	Challenging to create inclusive, consensus-led initiatives in regions that often have weak governance; for additionality, must go beyond high/low-risk rating systems; need committed buyers to support jurisdictional sourcing and expand beyond a small number of target landscapes	Requires certification and/ or transparent monitoring and evaluation to be externally demonstrable	Amazon Soy Moratorium, RSPO jurisdictions; SourceUp initiative links agri-commodity companies with multi-stakeholder initiatives in producing regions

### Direct sourcing

Where companies face sustainability risks, they may seek to simplify their supply chains and increase direct sourcing or vertical integration to have more control over the supply chain tier where risks occur (*37*, *38*). This shift is seen in the soy sector. As part of a multi-stakeholder initiative, the Soft Commodities Forum, traders have increased their direct sourcing in “priority” municipalities, where 70% of soy expansion into native vegetation takes place (table S2). Eliminating indirect sourcing is, however, not an effective, scalable, or necessarily equitable solution. Indirect suppliers are local actors, and efforts to eliminate them from supply chains exacerbate existing inequalities in global value chains (*39*). Commodity trading is otherwise dominated by a small number of mostly international companies who yield great market power. Although intermediaries are sometimes villainized as capturing value from farmers, in reality, their share of the global value chain is small. Across the cocoa supply chain, only 3 to 5% of net margins are captured by actors involved in the collection and export of cocoa, of which the local traitants will take only a share (*40*). In some cases, eliminating indirect sourcing is also physically impossible. The movement of cattle between farms is a feature of livestock farming in all parts of the world, as farms specialize in different parts of the cattle life cycle, whether cow-calf production or fattening.

### Cascading compliance

Cascading compliance approaches are where a focal company delegates responsibility for sustainable procurement to their direct (tier-1) suppliers, in the expectation that they, in turn, implement codes of conduct with their (“tier-2”) suppliers (*41*). Cascading compliance can be a sustainability multiplier, where tier-1 suppliers are required to ensure that their entire supply base meets sustainability standards, not just the part delivering products to the focal company. One risk of cascading compliance approaches is that focal companies pass on the burden of enforcing sustainable procurement onto potentially lower-capacity local suppliers (*42*). In practice, firms may choose hybrid approaches, engaging with or monitoring both direct and indirect suppliers (*43*, *44*). Palm oil traders making No Deforestation, No Peat and No Exploitation (NDPE) commitments, for example, not only take steps to educate supplier mills about these policies but also invest in building local capacity (e.g., farmer training in farm management and conservation) and use satellite monitoring to independently detect forest loss on their direct suppliers’ declared suppliers (*45*).

### Certification

By design, certification can address the challenges of indirect sourcing by providing standardized information that does not require the trader to monitor or know the producer and successive intermediaries. The penetration of certification varies across commodities—from close to zero for cattle products to 23 to 38% of global cocoa—with uncertainty around the exact figures because of products being double or triple certified by competing standards (*46*). Certification labels allow brands to communicate the sustainability credentials of products directly to consumers, although evidence of the effectiveness of certification is mixed (*47*). Certification has the potential to include smallholder farmers into sustainable procurement initiatives, but access to certification is not uniform, and the additionality of certification depends on the context and is not guaranteed (*48*). Globally, certified farms are disproportionately located in places with good market access, not necessarily where they are needed for biodiversity conservation and poverty alleviation (*49*). Many certification programs struggle to reach independent smallholders not organized into cooperatives or company schemes (*50*). The positive impacts of certification may also be undermined by other changes in the surrounding landscape. Certification under the Roundtable for Sustainable Palm Oil (RSPO) has reduced illegal deforestation on certified plantations but had little impact on overall deforestation rates as farmers clear more in other areas (*51*). Similarly, there is no evidence that RSPO certification has reduced fire or peatland clearance (*51*, *52*). Arguably, many of the challenges of certification stem from price premiums being set too low. Certification still mostly relies on cost-minimizing mass-balance or book-and-claim systems, where certified products are mixed with noncertified products or divorced from actual product flows entirely. Under book-and-claim systems, downstream companies pay a premium depending on how much certified product they want to buy, but they do not know to what degree the products they procure are actually compliant with their sourcing standards.

### Traceability

Many companies are investing heavily in efforts to improve traceability back to farm, both to increase knowledge of their supply chain and assess the risks associated with their procurement. In 2020, the palm oil trader Musim Mas, for example, reported 66% traceability back to the plantation for its fresh fruit bunches, up from 49% in 2019 (*53*). Traceability is inherently easier for direct than for indirect sourcing. The Brazilian soy trader Amaggi reports 100% traceability to farm for its direct sourcing but only 22% traceability for its indirect sourcing (*54*). For indirect sourcing, traceability may require mapping back to more producers than a company actually sources from, given the lack of segregation in most commodity supply chains. In many contexts, particularly where the supply chain is not formally organized, traceability efforts are only just starting and will be challenging to scale up. Under the CFI, for example, participating companies have so far mapped only a fraction (<46%) of their supply chain (fig. S3). The reliability of farm-level mapping efforts is uncertain because of their partial coverage and fluidity of trading relationships. So far, companies in the CFI have only mapped farms registered in cooperatives, although membership of cooperatives is fluid, with farmers signing up and dropping out seasonally (*55*). In addition, most farmers have two to three cocoa plots, although they usually only report a single one to the trading companies and it is not unheard of for cooperatives to “top up” their production with cocoa sourced from nonmember farmers (*56*).

Given the costs and challenges of improving traceability, some companies have limited efforts to high-risk regions. This is in practice what soy traders are doing as part of the Soft Commodities Forum, which focuses on sourcing in 61 priority municipalities in Brazil. In focusing on a subset of the supply chain, hot spot approaches inevitably ignore risks in other regions. As much as one-third of soy-associated vegetation clearing in the Cerrado occurs outside the Soft Commodities Forum’s priority municipalities (*57*). Hot spots are also a moving target—the deforestation frontier is dynamic and constantly shifting (*58*). This requires governance efforts to also be dynamic—ideally proactive—in identifying landscapes where the implementation of sustainable sourcing standards is prioritized.

Last, traceability must also be externally demonstrable. Companies may use third-party audits for external validity of their traceability efforts, but these audits often only include a fraction of the supply chain. Minerva’s audits of their cattle procurement in the Amazon cover only 10% of their purchases (*59*), and allegations of noncompliant sourcing continue (*60*). These concerns can be addressed through transparency—where companies make public data about their supply chains.

### Transparency

Corporate disclosures and transparency allow corporate claims of traceability and good production practices to be verifiable and discourage greenwashing (*3*, *61*, *62*). The information that companies disclose, however, is rarely aligned with reporting norms such as the Accountability Framework initiative—a consensus-based framework for the type and quality of supply chain information that companies should report (*63*). This can be seen from the variety of data sources required in this study to piece together each trader’s direct and indirect sourcing (Table 2), despite the Accountability Framework’s specification that “companies should report on the proportion of supply chain volume that is traceable to specific direct or indirect suppliers at each supply stage” (*63*). Transparency is most powerful where a norm is established, and supply chain information disclosed across an entire sector. If traceability and transparency are optional and implemented only by lead firms, as is currently the case (*64*), then it risks market bifurcation, whereby products with traceable and transparent supply chains cover only a subset of the market. Although some supply chains may be “clean,” such partial efforts can fail to deliver net positive impacts, as noncompliant products are simply diverted into less discerning parts of the market (*65*).

**Table 2.** Data sources. Summary of data sources used to quantify the prevalence of indirect sourcing in each commodity context.

**Context**	**Data sources used to quantify** **the prevalence of indirect** **sourcing**
South American soy	Trase data on traded volumes; corporate reports; Soft Commodities Forum progress reports
Cocoa in Côte d’Ivoire	Trase data on traded volumes; company self-disclosures of the location and size of supplier cooperatives; cocoa production per cooperative estimated from the number of cooperative members and farm production data
Indonesian palm oil	Trase data on traded volumes; corporate reports; capacity of individual processing facilities
Live cattle in Brazil	Trase data on traded volumes; animal transport permits

Governments can play a key role in facilitating a level playing field by either mandating company disclosure or facilitating access to key datasets on supply chains to help reveal direct and indirect sourcing patterns. In Brazil, the animal movement permits used in this study to track indirect sourcing are public documents according to legal assessments (*60*), although government ministries have moved to discourage their use (*66*). Access to property ownership information from the Brazilian rural cadaster—a key resource for monitoring and tracing commodities back to farm—is also variable from state to state. In Côte d’Ivoire, the coffee and cocoa board, CCC, operates an online database for tracking production from the first buyer (the cooperative or pisteur/traitant) to the port, and a paper-based system of receipts for tracking cocoa from each farmer to the first buyer. Making the database public would allow all trading companies to trace their cocoa back to local regions (“sous-prefectures”); digitizing and publishing the system of receipts would facilitate complete traceability all the way back to farmers for all companies regardless of their resources (*56*). The coffee and cocoa board are also actively GPS mapping the locations of cocoa farms (*67*). Who will have access to these data is not yet clear, but making these types of data public would remove the burden from individual companies and sidesteps companies’ concerns about releasing competition-sensitive data. It allows collaboration in problem-solving. All companies can see where their sourcing overlaps and build coalitions that can reduce costs by working together (*68*).

Regardless of the level of transparency, not all commodity-driven deforestation can be linked to commodity supply chains using available data. Illegal commodity production is notoriously hard to trace. In Côte d’Ivoire, for example, 18% of cocoa is grown inside protected areas (*69*), although few companies admit to purchasing this cocoa, much of which enters their supply chains through indirect sourcing. There can also be a temporal mismatch between deforestation and supply chain monitoring. Cocoa and oil palm, for example, take 2 to 5 years to first harvest. Areas are cleared speculatively by “future farmers”—most of the expansion of cocoa farms in Côte d’Ivoire, for example, is linked to recent migrants (*70*), who are not reached by ongoing corporate sustainability programs and not included in current traceability efforts (*50*). Data quality also limits accountability at the farm level. In cattle supply chains in the Brazilian Amazon, state-of-the-art supply chain mapping is only able to track the purchase and sale of cattle from properties responsible for 29% of deforestation and 50% of the pasture area (*9*). Mismatches in property ownership information and cattle movement records mean that the remaining 71% of deforestation and 50% of pasture cannot be linked to specific companies’ supply chains (*9*). Addressing these sources of deforestation requires looking beyond supply chains, working at the landscape level.

### Landscape and jurisdictional approaches

Commodity-centric landscape governance efforts are multi-stakeholder initiatives addressing sustainability issues associated with commodity production within specific landscapes (biomes or subnational jurisdictions) (*71*). Across our focus contexts, landscape approaches include the Amazon Soy Moratorium, Cerrado Working Group, Soft Commodity Forum’s priority municipalities, RSPO’s jurisdictional approach, and the CFI’s priority regions. By having a lens larger than a specific supply chain and incorporating all actors and land uses within the focal area, landscape approaches offer the potential to “internalize” systemic challenges such as hard-to-trace indirect or illegal sourcing and the drivers of deforestation over which supply chain–focused approaches have limited reach, such as speculative land clearing (*50*, *72*). Landscape approaches can thereby reduce costs and redundancies of investing in farm-level traceability, transparency, and certification. Although landscape approaches have the advantage of being able to find solutions across commodities, all too often they are focused on only one target commodity. Among the Soft Commodity Forum’s priority municipalities, for example, only 21% of native vegetation loss is for soy (*73*). The other 79% is converted to other uses—notably cattle ranching. Approaches that bring together the cattle and soy sectors will be key in reducing intercommodity leakage and breaking the link between agriculture and deforestation (*2*).

To be successful, landscape approaches require buy-in from farmers, financial commitment from companies, and support from local or national government. The business case for sustainable commodity production can be made at a local level by including a strong focus on the welfare of local communities alongside the monitoring of sustainability risks such as deforestation. Companies can provide financial incentives through “jurisdictional sourcing” efforts, where they commit to preferential sourcing from verified sourcing areas—jurisdictions that implement time-bound landscape conservation plans (*74*–*76*). Jurisdictional sourcing efforts are highlighted by the SourceUp initiative (*75*), but committed buyers remain few and far between. Notably, the Consumer Goods Forum’s Forest Positive Coalition of Action, a collection of the world’s largest brands (including Carrefour, Mars, Mondelēz, Nestlé, and Unilever), has identified landscape approaches as one of four key pillars of their sustainable sourcing efforts, alongside trader engagement, transparency and accountability, and government and stakeholder engagement (*77*). Their annual investment in landscape programs amounts, however, to only 9 million dollars (*77*)—a sum less than 0.0128% of the annual profit of participating companies (table S7).

### Including indirect suppliers and combining interventions in sustainable procurement

The corporate sustainability agenda, accelerated by the emergence of importer-country due diligence legislation, presents a fundamental challenge to traditional commodity trading, which has historically focused on financial and logistic considerations, and much less so on where products came from and how they were produced (*22*). This study shows that indirect sourcing is prevalent if not dominant in many key agricultural commodity supply chains, constituting a sizable blind spot for traders’ monitoring and sustainability commitments.

We focus here on major forest-risk commodities, although our insights are relevant also to other supply chains and sustainability issues. The patterns that make indirect sourcing common (commodities traded in bulk originating from large numbers of often small-scale producers) apply to other agricultural commodities such as coffee, rice, rubber, and orange juice concentrate, and sectors including mining or apparel (*73*, *74*). Moreover, indirect sourcing presents a sustainability blind spot not only for traders but also for downstream buyers. Across 449 publicly listed companies in the food, textile, and wood products sectors, where companies have supplier codes of conduct, these are in most cases (60.5%) limited to their direct suppliers (*78*). Indirect suppliers have similarly been identified as a disproportionate source of risk exposure for companies in the automotive, electronics, pharmaceutical, and consumer goods sectors (*79*). Addressing deforestation in indirect supply chains already poses a notable challenge; the problem becomes even greater for issues such as pesticide contamination or social issues that cannot be mapped using broad-coverage earth observation data (*42*). This imbalance is seen in the published grievances against palm oil companies—where social issues, including wage disputes, forced labor, and violence—are underreported relative to deforestation (*80*).

Despite more than a decade of corporate sustainable sourcing commitments, commodity-driven deforestation continues (*1*, *2*). To deliver on promises to eliminate deforestation from commodity supply chains, sectoral sustainability initiatives, such as the CFI, Amazon Soy Moratorium, and Cattle Agreements, need to acknowledge, monitor, and report on indirect sourcing—and ultimately ensure that it does not remain a barrier to delivering on sustainability goals. The on-the-ground reality of commodity production—widespread indirect sourcing, smallholder production, informal supply chain relationships, and multiple interacting drivers of deforestation in commodity-producing landscapes—means, however, that no single intervention is sufficient to break the link between commodity production and deforestation. Rather, there needs to be a broadening of responsibility for ensuring greater transparency and accountability—as well as more equitable sharing of benefits—across the supply chain, including indirect sourcing. Interventions by multiple actors are needed to help achieve this. Efforts to trace commodities from farm to fork should be enabled by producer government policies that prioritize transparency and unlock data on supply chains. Trading companies must invest in sustainable procurement efforts that extend beyond their own direct supply chains, and policies from consumer countries, such as on mandatory due diligence, need to account for the prevalence of indirect sourcing and informal production and trade. Only by layering these policies with inclusive land use governance, rule enforcement, green finance, and other corporate sustainability efforts (*78*) may we create the necessary mix of incentives for sustainable development within production landscapes.

## MATERIALS AND METHODS

### Quantifying the prevalence of indirect sourcing

We used detailed shipping data from 2018 to 2019 from the Trase initiative (https://trase.earth/) to identify the commodity trading companies handling the top 60% of exports of our four focus commodities: soy from South America (2018 data on trader market share across Brazil, Argentina, and Paraguay), cocoa from Côte d’Ivoire (2019), palm oil from Indonesia (2018), and live cattle exports from Brazil (2019). These data include exports both as a raw commodity (e.g., cocoa beans) and as processed products (e.g., cocoa butter or paste). When reporting market share per trader, all volumes were converted into “raw product equivalents” (e.g., the volume of cocoa beans required to produce cocoa butter exports) by multiplying these product volumes by standard conversion coefficients (*79*).

We reviewed corporate annual, sustainability, and traceability reports to identify and extract the percentage of each trader’s sourcing that was direct and indirect in each producer country, and the identity of the direct and indirect suppliers (whether their own plantations or farms, etc.). Where these figures were not reported, we used context-specific supply chain data to estimate direct and indirect sourcing (Table 2). Details of the assessments for each commodity are provided below.

#### South American soy

Several soy traders report the proportions of direct and indirect sourcing for only part of their supply chain: Bunge only for priority municipalities included in the Soft Commodities Forum, Viterra only for the Cerrado, and COFCO only for Mato Grosso and Matopiba. The percentages sourced directly/indirectly were then combined with Trase’s trade data (i.e., exported volumes in per region) to show the overall sourcing patterns. The remaining volumes were recorded as “unknown.” In corporate disclosures, traders do not differentiate between soy sourced via cooperatives and other aggregators. In Fig. 2, we therefore use a color gradient to reflect this uncertainty in the type of indirect sourcing used. Where trading companies have undergone a name change, we report the current name (i.e., “Glencore Agriculture” is named as Viterra throughout the article).

#### Cocoa from Côte d’Ivoire

Trading companies (and cocoa processors) do not directly disclose the volumes sourced from cooperatives or other local intermediaries, although many do disclose which cooperatives they buy from, and the number of farmers per cooperative (Supplementary Text). For each cooperative, we estimate the volume sold to their customer traders by multiplying the number of reported member farmers by cocoa production per farm. For 440 of 710 cooperatives linked to a known trading company, the number of supplying farms was reported by the trading company. For the remaining 270 cooperatives, the number of farmers was estimated by taking 1000 Monte Carlo samples (with replacement) from the known cooperative sizes (median: 591 farmers per cooperative; mean: 766 farmers per cooperative). Production per farmer (ton/year) was also estimated using Monte Carlo resampling from production data from 441 cocoa farmers across Côte d’Ivoire (*81*). The Monte Carlo simulation produced relatively narrow confidence intervals (fig. S2), and so, we focus on the mean values per trader in this article. More details on the uncertainty, data sources, and methods are available in (*79*). Last, the percentage of cocoa purchased via cooperatives (localized indirect sourcing) was calculated as the sum of the cocoa bean sourcing from each cooperative, divided by the trader’s total procurement in Côte d’Ivoire. Two of our focal traders, S3C and Africa Sourcing, do not disclose their cooperative suppliers, and so, their sourcing is listed as unknown.

We also estimate the percentage of each trader’s cocoa sourcing that has been mapped to farm level. We extracted the number of farms mapped from statistics listed in each company’s 2019/2020 CFI reports. This was again converted to a cocoa volume using Monte Carlo sampling of farmer production data. Where trading companies reported farms mapped both “through direct investment” and “on behalf of clients,” we used the sum of these two figures as the total number of cocoa farms mapped in Côte d’Ivoire.

#### Indonesian palm oil

All assessed palm oil traders provide information on the percentage of products sourced from their third-party and own-company mills, though in a nonstandardized manner. Sinar Mas reports that 39% of its sourcing comes from its own mills (*82*), of which 70% is from “nucleus plantations” (vertically integrated direct sourcing), 20% from “plasma smallholders” (direct sourcing), and 10% from “independent smallholders & third party producers” (localized indirect sourcing) (*83*). The remaining 61% comes from third-party mills. To conservatively estimate indirect sourcing through third-party mills, we assumed that all palm oil sourced via third-party mills that was traceable to plantation came from mills’ own plantations (i.e., was direct sourcing) rather than originating from independent smallholders or other aggregators. This assumption is based on the fact that fresh fruit bunches sourced from a mill’s own plantation are more readily traceable (mills are often located on site) than fruit sourced via intermediaries. Traceable volumes are extracted from each refinery’s traceability summary reports, and the overall proportion traceable was calculated by weighting the sourcing of each facility by its refinery capacity (table S4). As a check of the validity of using processing capacities to estimate sourcing volumes per facility, we verified that this method gave a similar estimate of sourcing via third-party mills to Sinar Mas’s self-reported numbers (57% versus 61%).

Musim Mas reports that “The bulk of our supply—90%—comes from external sources, meaning third-party mills outside Musim Mas control. These mills receive FFB either from their own plantations (equivalent to 40% of our supply) or independent smallholders (50%)” (*45*). Of the 10% of sourcing via their own mills, 70.2% comes from Musim Mas plantations, 3% from scheme smallholders, 0.1% from independent smallholders, and 26.7% from other third parties (*45*).

Wilmar reports each refinery’s sourcing (%) from Wilmar’s own mills and third-party mills, but they do not report overall volumes. We calculated volumes using each refinery’s processing capacity (table S4). In 2021, Wilmar also disclosed the origin of fresh fruit bunches for its own mills: 40.4% from Wilmar plantations, 4% from scheme smallholders, and the remaining 55.5% from third-party sources (*84*). For purchases through third-party mills, as above, we assumed that all traceable volumes sourced came from mills’ own plantations (direct sourcing), and untraceable volumes were sourced through other intermediaries (indirect sourcing).

When reporting the sourcing of Royal Golden Eagle, we focus on their subsidiary Apical Group, which operates refineries responsible for more than 86% of Royal Golden Eagles palm oil exports. In December 2020, Apical reported that they source 100% from third-party mills and that “29.4% is sourced from third party supplier mills’ own plantations while 70.6% is sourced from third party plantations” (*85*). Here, Apical counts Asian Agri (another company within the Royal Golden Eagle conglomerate) as a third-party supplier. Because Apical does not report what percentage of its direct sourcing comes from Asian Agri mills, in Fig. 2, we use a color gradient when reporting their direct/vertically integrated sourcing.

#### Brazilian live cattle exports

The market share of live cattle traders was calculated from Trase’s shipping data. For 20% of trade, the trading company was not listed; market shares were therefore calculated on the basis of the remaining 80% of trade where the trader was known. We quantified each trader’s indirect sourcing as the percentage of their cattle purchases (2017–2019) that came from direct suppliers who, in turn, bought in cattle. For this, we used a database of cattle movements, known as the Guia de Trânsito Animal (GTA). The GTA is a legally required document detailing the movement of batches of cattle between properties in Brazil, including the date of each movement, the identity of the farm, slaughterhouse, or trader sending and receiving cattle, and the number of cattle. Together, the dataset used in this analysis includes 33 million records of cattle movements across 23 states in Brazil, processed and cleaned according to (*12*).

Direct suppliers were identified as the farms moving cattle to a property owned by the trader (identified by the first eight digits of the CNPJ (Cadastro Nacional da Pessoa Jurídica), which is unique to each company; table S5). Direct sourcing (i.e., purchases from “complete cycle” cattle farms that do not buy in cattle but produce and rear their own cattle) was classified using a cutoff of 20 cattle bought in across 2017–2019. If a property bought in 20 or fewer cattle, it was considered as part of the trader’s direct sourcing. This cutoff allows for small-scale movement of cattle for purposes such as breeding. For comparison, the average herd size in the state of Pará, the main source of live cattle exports, is 148 cattle per property (*16*). Our results are not sensitive to this cutoff (table S6). In cases where farmers both produce calves on farm (complete cycle) and buy in some cattle, where their purchases exceed our 20 cattle threshold, all cattle from that property are considered as being in the indirect supply chain. There is no widely adopted system for tracking individual cattle in Brazil, and so, from a trader’s perspective, when they purchase from a direct supplier, they do not have any mechanism of assessing whether the cattle was born on that farm or elsewhere. Similarly, although traders could map the spatial location of their direct suppliers, we do not, however, count their indirect sourcing as “localized,” because direct suppliers, in turn, source cattle from a wide region: 62 to 66% of cattle purchases are from municipalities other than where the direct supplier is itself located.

### Assessing deforestation risks in indirect sourcing

To illustrate how deforestation risks are distributed across direct and indirect sourcing, we present analyses for cocoa from Côte d’Ivoire and Brazilian live cattle exports. Similar calculations are not possible for palm oil and soy traders because data are not available to spatialize where companies source directly versus indirectly. To do so would require soy traders to disclose the locations and volumes of their supplier farms and silos, and palm oil traders to disclose for each of their supplier mills the mix of supply that come from mills’ own plantations, rather than smallholders or local aggregators.

#### Cocoa from Côte d’Ivoire

We estimate the deforestation risk of cocoa traders based on the cocoa-driven deforestation in the departments from which they sourced. This calculation requires three steps: first, identifying the volumes sourced by traders from each department through their localized and indirect supply chains; second, identifying the area of cocoa-driven deforestation (in hectares) per department; and third, the production of cocoa (in kilotons) per department. Step one is explained above; steps two and three are discussed in more detail below.

Cocoa-driven deforestation (in hectares) was identified by intersecting remote-sensing products of cocoa (*69*) and forest loss (*17*) to identify the area of cocoa production in 2019 that was planted on land that was converted between 2000 and 2015. The cocoa land cover map was 20-m^2^ resolution, based on Sentinel-1 and Sentinel-2 satellite images. The land cover map was based on 30-m^2^ resolution Landsat imagery, processed by the Food and Agriculture Organization of the United Nations (FAO) and Côte d’Ivoire’s BNETD (National Bureau of Technical Studies and Development) as part of the monitoring efforts for the implementation of REDD+ programs in the country. Hence, we use an allocation period of 15 years (forest cleared between 2000 and 2015) and a time lag of 4 years (2015–2019) to allocate deforestation risk to cocoa harvested in 2019. This time lag reflects that land cleared in 2015 (or earlier) and planted with cocoa trees takes 3 to 5 years to become productive. The total area of cocoa-driven deforestation for Côte d’Ivoire was 289.1 kha (fig. S1).

The area of cocoa-driven deforestation per department was then divided by cocoa production (in kilotons) to estimate the deforestation risk per department (ha/kton), which was linked to trader flows. Because there are no official, publicly available subnational data on cocoa production in Côte d’Ivoire, cocoa production per department was calculated using the remote-sensing map of cocoa area and a cocoa suitability map (*86*) to weight the cocoa area in each department by its relative suitability for cocoa production.

The “relative suitable area” (RSA) for cocoa in each department was calculated as

$${\text{RSA}}_{d}=\sum_{n=1}^{i}{\text{area}}_{n}*{\text{suitability}}_{n}$$

where *d* represents all departments, *n* represents all pixels under cocoa, area*_n_* represents the area covered by each pixel, and suitability*_n_* represents the suitability index (0 to 1) of each pixel.

Cocoa production (in tons) per unit of RSA was calculated as

$$\text{pRSA}=\frac{{\text{production}}_{\text{ICCO}}}{\sum_{d=1}^{j}{\text{RSA}}_{d}}$$

where production_ICCO_ is the total cocoa production volume (kg) of Côte d’Ivoire (from International Cocoa Organization, ICCO, statistics). Last, we calculated cocoa production per department

$${\text{production}}_{d}={\text{RSA}}_{d}*\text{pRSA}$$

The confidence intervals in the deforestation risk of each trader’s sourcing (Fig. 4) reflect the uncertainty in the estimation of volumes sourced by each trader from each cooperative.

#### Brazilian live cattle exports

We crossed lists of each trader’s suppliers against properties embargoed by the Brazilian environmental enforcement agency, Ibama, to identify risks among traders’ direct and indirect suppliers. Properties are embargoed by Ibama for environmental crimes—illegal deforestation, preventing reforestation, and other acts including illegal hunting, polluting, and building without permission in natural areas. We focus on embargoes as a simple demonstration of sustainability risks in meat-packers’ direct and indirect supply chains. Embargoes are, however, only the tip of the iceberg—less than 1% of the Amazon’s illegal deforestation goes on to be embargoed (*87*).

We identified cattle movements from embargoed properties as cases where (i) the tax identifier [Cadastro de Pessoas Físicas in Portuguese (CPF)] of the property transferring cattle in the GTAs matched a CPF in the embargo list, (ii) the property was listed in the same municipality as the embargo, and (iii) the transport date in the GTAs came after the date that the property was placed on the embargo list (the “Data de Inserção na Lista,” in Portuguese). We then report the number of embargoed suppliers as a percentage of each trader’s suppliers for which the CPF was listed in the GTA data (Supplementary Text).

## Notes

View/request a protocol for this paper from *Bio-protocol*.
