# Supplementary material for: Addressing indirect sourcing in zero deforestation commodity supply chains
Source: Sci Adv. 2022 Apr 29;8(17):eabn3132. doi: 10.1126/sciadv.abn3132 (PMC9054003; doi:10.1126/sciadv.abn3132)
Supplement: Supplementary file 1 — Supplementary Text Figs. S1 to S4 Tables S1 to S7 [file sciadv.abn3132_sm.pdf]

Science Advances
AAAS

Supplementary Materials for

Addressing indirect sourcing in zero deforestation commodity supply chains

Erasmus K. H. J. zu Ermgassen*, Mairon G. Bastos Lima, Helen Bellfield, Adeline Dontenville, Toby Gardner, Javier Godar, Robert Heilmayr, Rosa Indenbaum, Tiago N. P. dos Reis, Vivian Ribeiro, Itohan-osa Abu, Zoltan Szantoi, Patrick Meyfroidt

*Corresponding author. Email: erasmus.zuermgassen@uclouvain.be

Published 29 April 2022, *Sci. Adv.* **8**, eabn3132 (2022)
DOI: 10.1126/sciadv.abn3132

This PDF file includes:

Supplementary Text
Figs. S1 to S4
Tables S1 to S7

Supplementary Text

Data availability

The data and code required to reproduce statistics and figures reported in the manuscript are available in Zenodo (10.5281/zenodo.6038988).

South American Soy: variable transparency

Discussions around the sustainability of soy in South America have tended to focus on deforestation in the Brazilian Amazon (1), though soy is a driver of forest loss across the continent (2). In line with this focus, while all (7/7) traders disclosed some information about direct and indirect sourcing within Brazil, 5/7 traders sourcing soy in Argentina and 4/6 traders sourcing soy in Paraguay do not provide similar information for these contexts. Sourcing is marked 'unknown' in these cases.

Mechanisms of indirect suppliers' deforestation risk

The elevated deforestation risk among indirect suppliers can arise through multiple processes. First, they may reflect intrinsic differences in the characteristics (including land management and agricultural practices) of farmers who sell directly to traders, through cooperatives, or via other local intermediaries. Second, it may reflect the efficacy of sustainable sourcing initiatives which are focused on direct suppliers or cooperatives. Third, the exclusion of indirect suppliers creates a loophole where risks are not reduced among indirect suppliers. Lastly, the focus on direct suppliers also creates the opportunity for leakage, where non-compliant production is displaced from direct to indirect suppliers.

Risk of cattle purchases from embargoed properties/holdings

We crossed lists of each traders' suppliers against properties embargoed by the Brazilian environmental enforcement agency, Ibama, to identify risks among traders' direct and indirect suppliers. Our method treats (multiple) properties linked to the same CPF in the same municipality as a single holding. These properties are often adjacent - according to (12), 40% of multi-property cases are located less than 0.01 km apart, with a median distance between properties of 0.4 km. In practice, farmers manage their cattle across these properties as a single herd, though when monitoring their zero deforestation commitments, traders tend to treat each property separately. We therefore checked whether the embargoes we identify in traders' direct supply chains are cases where the embargoed area is on a sister property to the one listed as supplying cattle. We matched the supplier and embargo lists not only on the CPF and municipality (as above), but also the property name. 75.8% of our identified embargoes included a farm name, and of these, no embargoed areas were found on the *properties* directly supplying traders with zero deforestation commitments (Figure S4) – rather, embargoed areas were on other properties within the same holding. Among tier-1 indirect suppliers, embargoed areas were both detected on the properties (in 4-21% of cases) and holdings (in 79-96% of cases) selling cattle to the traders' direct suppliers. These results suggest that traders are successfully filtering out embargoes from their direct suppliers when assessed at the property-level, but not at the level of holdings, and that their monitoring is also not addressing embargoes among indirect suppliers, both at the property- and holding-level. Our findings mirror other work (12), also highlighting that traders' property-level monitoring creates a loophole for cattle from embargoed areas to enter their supply chains from their direct suppliers' sister properties within the same holding.

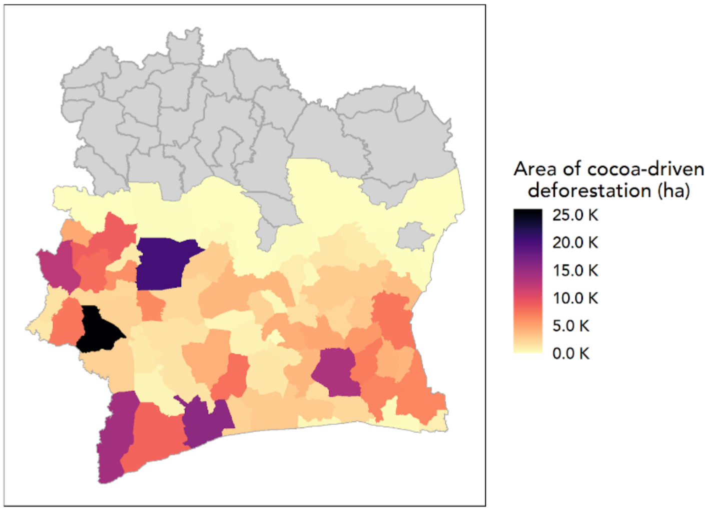

Fig. S1.
The area of cocoa-driven deforestation per département in Côte d’Ivoire, calculated as the area of cocoa in 2019 which was detected on areas of forest cleared between 2000-2015.

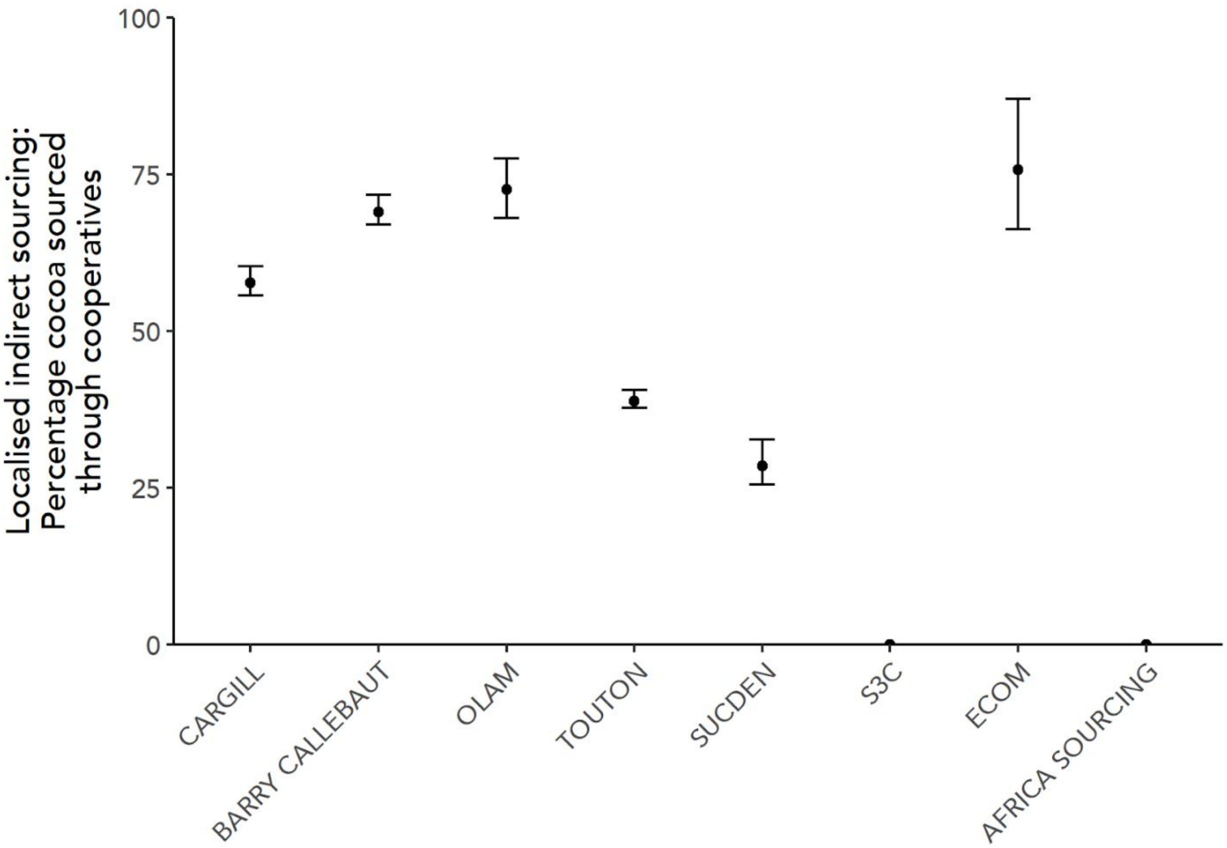

Fig. S2.
Mean estimate and 95% confidence intervals for the percentage of cocoa sourced from cocoa cooperatives (cf. traitants) for major cocoa traders in Côte d'Ivoire.

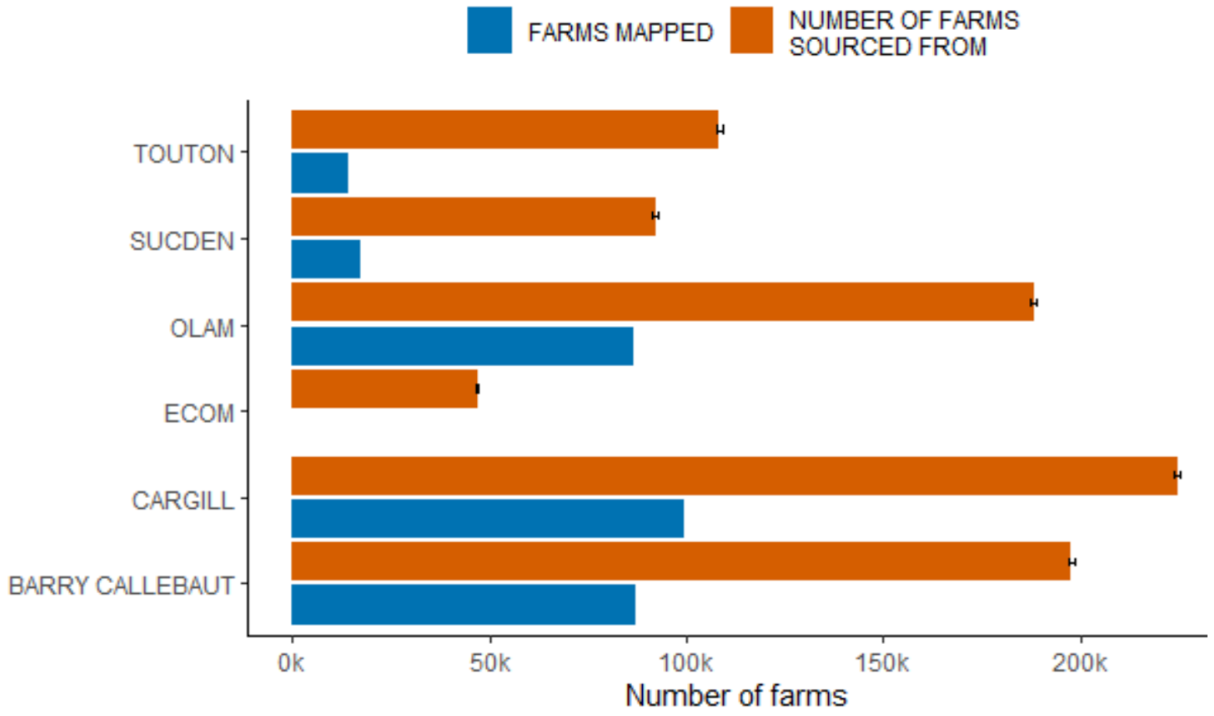

Fig. S3.
Comparison of the number of farms mapped in Côte d'Ivoire, as reported under the Cocoa & Forests Initiative, against Monte Carlo estimates of the number of farms in each trader's supply chain. Error bars are 95% confidence intervals. S3C and Africa Sourcing do not participate in the CFI and so no farm numbers are listed.

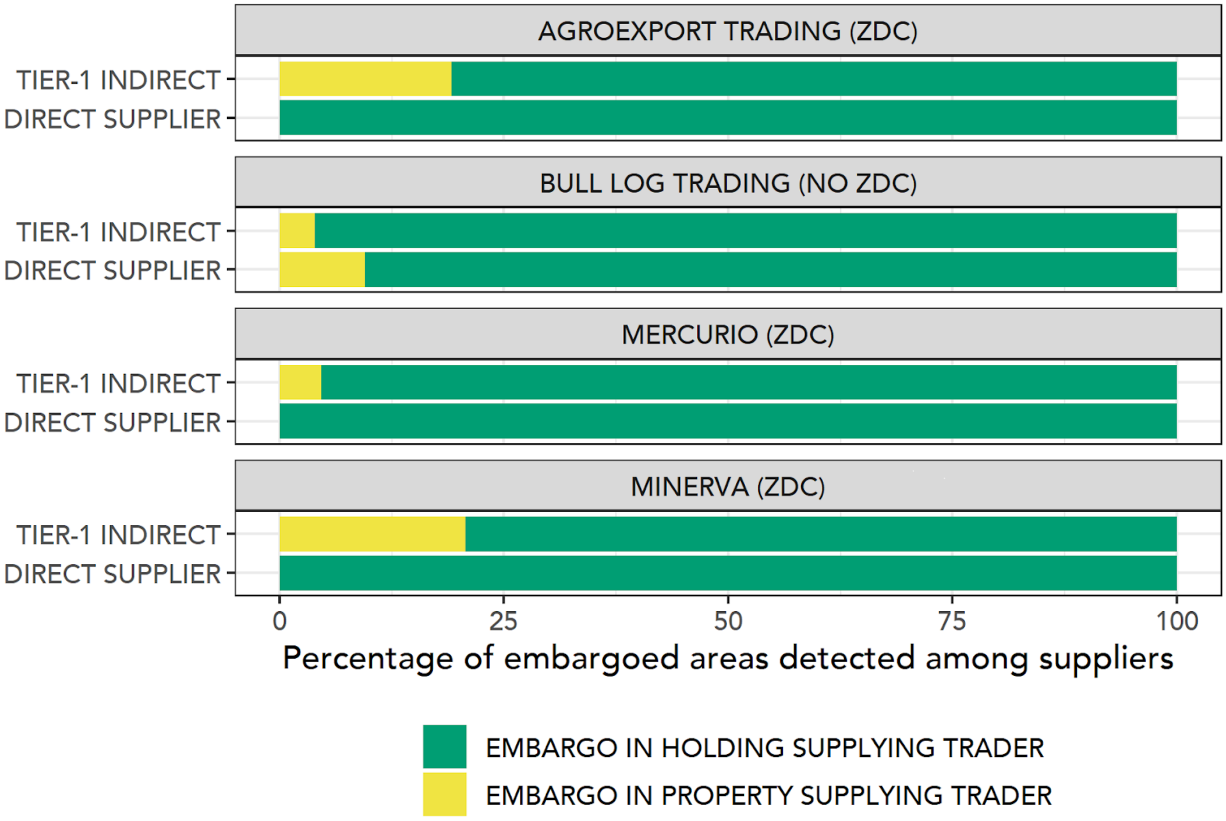

Figure S4.
For companies making a zero deforestation commitment (ZDC), all embargoed areas among their direct suppliers were detected within their direct supplier's holding (sister properties registered to the same owner, within the same municipality), rather than the specific property from which the trader purchased cattle. Among traders' tier-1 indirect suppliers (i.e. their direct suppliers' suppliers), 4-21% of embargoed areas were in the properties whose names match the name of properties from which the cattle movement was recorded.

Table S1.
How the terms ‘direct’ and ‘indirect’ suppliers or sourcing have been used in different commodity contexts. While in the soy sector, for example, indirect procurement is usually taken to mean any soy not purchased from the farmer who produced it, in crops produced by smallholders, such as cocoa or oil palm, the term ‘direct supplier’ has been used to refer to purchases from the first actor who aggregates products within a given local area - e.g. a farmer cooperative in cocoa, or a mill in oil palm (oil palm’s fresh fruit bunches are easily perishable and so are processed at local mills before being sold to other companies). In this manuscript we present a unified framework for direct and indirect sourcing (see main text). Note: in supply chain management, the separate term ‘indirect procurement’ is used to refer to purchases which do not add to a business's bottom line, e.g. buying office supplies or acquiring services.

Sector	Terminology used	Examples where this is referred to as such
Brazil beef	“Direct suppliers are farms which sell cattle directly to meatpackers, while indirect suppliers are farms which sell or transfer cattle to other farms or intermediaries.”	Grupo de Trabalho dos Fornecedores Indiretos na Pecuária Brasileira: https://gtfi.org.br/cadeia-da-carne-no-brasil/
Brazil soy	Indirect sourcing: soy not purchased directly from farmers, instead purchased from cooperatives or aggregators who operate silos (storage facilities) to which multiple farmers contribute soybeans.	Soft Commodities Forum progress report, December 2019: https://docs.wbcsd.org/2019/12/WBCSD_Soft_Commodities_Forum_progress_report.pdf.
Brazil soy	“Direct: soy sourced directly from a farmer.”<br>“Indirect: soy sourced from aggregators, cooperatives, and other third parties”	Louis Dreyfus company (2020) Soy Sustainability - Focus on Brazil & Argentina. Transparency Update: Sourcing Profile and Deforestation/Conversion Risks https://www.ldc.com/wp-content/uploads/Brazil-and-Argentina-Deforestation-Risk-Profile_24.6.2020_final.pdf
West Africa cocoa	The term “direct supply chain” is used to refer to cocoa beans purchased through cooperatives or producer groups.	Cocoa & Forests Initiative reports CFI reports,, e.g. https://www.worldcocoafoundation.org/wp-content/uploads/2018/08/WCF_Report_14.6_051420.pdf.
Indonesian Oil palm	Direct sourcing is where the mill or cooperatives (of smallholders) are known.<br><br>Indirect sourcing is where palm oil is purchased from local traders and refineries, or from independent smallholders, or “mills with whom we do not have	Cargill Supplier Engagement: https://www.cargill.com/sustainability/palm-oil/palm-supplier-engagement

	a direct commercial relationship”.	
Indonesian Oil palm	“Indirect mills listed represent mills supplied to Wilmar's third-party refineries, traders and/or bulkers.”	Wilmar Traceability Summary: https://www.wilmar-international.com/docs/default-source/default-document-library/sustainability/supply-chain/traceability-report-q3'-2019---q2'-2020/indonesia/mna-kuala-tanjung_201022.pdf?sfvrsn=2bb7b41d_2
Indonesian Oil palm	“Direct supply: sourced directly from third-party mills.”<br>“Indirect supply: procured from trading partners who in turn source oil from their own operations or third-parties.”	Bunge: https://www.google.com/url?sa=t&rct=j&q=&esrc=s&source=web&cd=&cad=rja&uact=8&ved=2ahUKEwjc5sb4trTuAhVN-6QKHTVIB7AQFjAAegQIBRAC&url=https%3A%2F%2Fbungeloders.com%2Fassets%2F2020-05%2FBunge_Sustainable_Palm_Oil_Sourcing_Policy.pdf&usg=AOvVaw3pRI044nQnar1EZHzh4Veg

Table S2.

Percentage of direct sourcing of the top soy traders in Brazil, comparing nationwide values vs. the initial 25 priority municipalities target as part of the Soft Commodities Forum (SCF). *Bunge only reports direct sourcing in priority municipalities (responsible for 23.4% of their soy sourcing in Brazil). COFCO reports their direct sourcing percentage only for Mato Grosso and Matopiba (70%), which together make up 39.6% of their soy sourcing in Brazil. Viterra reports their direct sourcing percentage only for the Cerrado (60.4%). The Cerrado makes up 42% of their soy in Brazil.

Company	% direct sourcing, across Brazil	% direct sourcing in 25 priority municipalities - June 2019 report	% direct sourcing in 25 priority municipalities - December 2020 report	Change in direct sourcing from 2019-2020 reports
Bunge	Not disclosed*	98%	98.4%	Increase
ADM	63	93.4%	88%	Decrease
Cargill	69	96.6%	97%	Increase
COFCO	Not disclosed*	84%	95.9%	Increase
Viterra	Not disclosed*	57.10%	64.9%	Increase
LDC	47	100%	100%	Increase
Amaggi	79	Does not participate in SCF	Does not participate in SCF	Not applicable

Table S3

Pixel classes used to identify cocoa-driven deforestation.

Code	Classification
221	Forest loss 2000-2015
212	Forest-agriculture transitions

Table S4.
The mill-level sourcing and capacity of Wilmar's palm oil processing facilities. Where a facility included statistics for crude palm oil, lauric acid, or kernel crushing separately, we report figures for crude palm oil. The figures for different products were similar and include overlapping mill suppliers. Source: (11).

Company	Processing facility	Mill type	Percent supply per processing facility	Capacity (MT/year)
Wilmar	PT Wilmar Nabati Indonesia, Padang	Own mills	25.98	858000
Wilmar	PT Wilmar Nabati Indonesia, Padang	Independent mills	74.026	858000
Wilmar	PT Wilmar Nabati Indonesia, Gresik	Own mills	5.79	3036000
Wilmar	PT Wilmar Nabati Indonesia, Gresik	Independent mills	94.21	3036000
Wilmar	PT Wilmar Nabati Indonesia, Balikpapan	Own mills	0.00	950000
Wilmar	PT Wilmar Nabati Indonesia, Balikpapan	Independent mills	100.00	950000
Wilmar	PT Wilmar Nabati Indonesia, Pelintung	Own mills	25.32	1980000
Wilmar	PT Wilmar Nabati Indonesia, Pelintung	Independent mills	74.67	1980000
Wilmar	PT Wilmar Cahaya Indonesia, Pontianak	Own mills	51.80	214500
Wilmar	PT Wilmar Cahaya Indonesia, Pontianak	Independent mills	48.19	214500
Wilmar	PT Sinar Alam Permai, Kumai	Own mills	1.80	990000
Wilmar	PT Sinar Alam Permai, Kumai	Independent mills	98.20	990000
Wilmar	PT Multi Nabati Sulawesi, Bitung	Own mills	0.00	594000
Wilmar	PT Multi Nabati Sulawesi, Bitung	Independent mills	100.00	594000
Wilmar	PT Multimas Nabati Asahan, Pulo Gadung	Own mills	0.00	82500
Wilmar	PT Multimas Nabati Asahan, Pulo Gadung	Independent mills	100.00	82500
Wilmar	PT Sinar Alam Permai, Palembang	Own mills	27.30	693000
Wilmar	PT Sinar Alam Permai, Palembang	Independent mills	72.70	693000

Wilmar	PT Multimas Nabati Asahan, Kuala Tanjung	Own mills	12.71	1914000
Wilmar	PT Multimas Nabati Asahan, Kuala Tanjung	Independent mills	87.29	1914000
Wilmar	PT Multimas Nabati Asahan, Paya Pasir	Own mills	0.00	450000
Wilmar	PT Multimas Nabati Asahan, Paya Pasir	Independent mills	100.00	450000
Wilmar	PT Wilmar Nabati Indonesia, Dumai	Own mills	26.53	1353000
Wilmar	PT Wilmar Nabati Indonesia, Dumai	Independent mills	73.47	1353000

Table S5.
To identify the network of farms supplying each trader, we queried animal movement data (GTA) based on the suppliers to the following business registry numbers (CNPJ, Cadastro Nacional da Pessoa Jurídica in Portuguese), which were used to handle live cattle exports by each company.

Trader	CNPJs assessed
Minerva Global Foods	67620377000890
Agroexport Trading	25333824000538, 25333824000376, 25333824000457, 25333824000961
Bull Log Trading	20819178000176, 20819178000338
Mercurio Alimentos	11831785000160

Table S6
The percentage of live cattle traders' sourcing which is classified as 'direct', depending on the cut-off used to define direct sourcing: either <20 or <100 cattle may be bought by a direct supplier, before it is considered part of the indirect supply chain.

Trader	Max 20 cattle bought by traders' direct suppliers	Max 100 cattle bought by traders' direct suppliers
Minerva Global Foods	2.55%	3.59%
Agroexport Trading	3.62%	6.48%
Bull Log Trading	6.03%	12.10%
Mercurio Alimentos	1.15%	2.77%

Table S7

The 2020 profit of companies involved in landscape initiatives, as listed in the Consumer Goods Forum's Forest Positive Coalition of Action 2021 annual report. We contrast the $70.6 billion that these companies declared in profit against the $9 million they invested in landscape programs (ca. 0.0128% of annual profit). This number excludes figures for Mars, for whom no public data on profit are available, because they are a privately-owned company. CR = conversion rate to USD, based on currency values in January 2022.

Company	Net Profit	Currency	CR	USD (billions)	Source:
Carrefour	6.41E+08	EUR	1.13	0.72433	https://www.retaildetail.eu/en/news/food/carrefour-sees-record-performance-2020
Colgate-Palmolive	1.02E+10	USD	1	10.17	https://www.macrotrends.net/stocks/charts/CL/colgate-palmolive/gross-profit
Danone	2.23E+09	USD	1	2.234	https://www.macrotrends.net/stocks/charts/DANOY/danone/net-income
General Mills	3.14E+09	USD	1	3.14	https://www.statista.com/statistics/261177/operating-profit-of-general-mills-worldwide/
Grupo Bimbo	9.11E+09	MEX	0.047	0.42817	https://www.statista.com/statistics/796990/net-income-bimbo-worldwide/
Jerónimo Martins	3.12E07	EUR	1.13	0.35256	https://econews.pt/2021/03/03/jeronimo-martins-posts-a-net-profit-of-e312m-in-2020/
Mars	NA	USD	1	NA	
Mondelez	1.04E+10	USD	1	10.466	Net revenue: https://ir.mondelezinternational.com/news-releases/news-release-details/mondelez-international-reports-q3-2021-results
Nestlé	1.22E+10	CHF	1.09	13.298	https://www.statista.com/statistics/268889/net-profit-of-the-nestle-group-worldwide/

PepsiCo	7.00E+09	USD	1	7	https://www.statista.com/statistics/242288/global-net-income-of-pepsico/
Reckitt	1.20E+09	GBP	1.33	1.59999	https://finbox.com/LSE:RKT
Tesco	9.73E+08	GBP	1.33	1.29409	https://www.tescoplc.com/investors/reports-results-and-presentations/financial-performance/five-year-record/
Unilever	6.38E+09	USD	1	6.375	https://www.macrotrends.net/stocks/charts/UL/unilever/net-income
Walmart	1.35E+10	USD	1	13.51	https://www.macrotrends.net/stocks/charts/WMT/walmart/net-income
